# Dialysis for older adults: why should the targets be different?

**DOI:** 10.1007/s40620-023-01835-1

**Published:** 2024-01-05

**Authors:** Priyanka Khatri, Andrew Davenport

**Affiliations:** 1 Fast and Chronic Programmes, Alexandra Hospital, Queenstown, Singapore; 2 Yong Loo Lin School of Medicine, National University of Singapore, Singapore, Singapore; 3 UCL Department of Renal Medicine, Royal Free Hospital, University College London, Rowland Hill Street, London, NW3 2PF UK

**Keywords:** Incremental haemodialysis, Elderly, Kt/Vurea, Fistula, Dialysis catheter, Nutrition

## Abstract

The number of patients aged > 75-years treated by dialysis continues to increase, particularly in developed countries. Haemodialysis is a well-established treatment with national and international clinical guidelines designed to provide patients with optimal treatment. However, these were developed when the dialysis population was younger, and less co-morbid. This change in patient demographics questions whether these guideline targets still apply to older patients. More patients now start dialysis with residual kidney function and could benefit from a less frequent dialysis schedule. Older patients have a lower thirst drive, so lower interdialytic gains, reduced appetite, muscle mass and physical activity would potentially allow starting dialysis with less frequent sessions a practical option. Similarly, patients with residual kidney function and lower metabolic activity may not need to meet current dialyser Kt/Vurea clearance targets to remain healthy. Instead, some elderly patients may be at risk of malnutrition and might need liberalisation of the low salt, potassium and phosphate dietary restrictions, or even additional supplements to ensure adequate protein intake. Although a fistula is the preferred vascular access, a forearm fistula may not be an option due to vascular disease, while a brachial fistula can potentially compromise cardiovascular reserve, so a dialysis catheter becomes the de facto access, especially in patients with limited life expectancy. Thus, clinical guideline targets designed for a younger less co-morbid dialysis population may not be equally applicable to the older patient initiating dialysis, and so a more individualised approach to dialysis prescription and vascular access is required.

## Introduction

The demographics of the end-stage kidney disease (ESKD) population are changing, especially in developed countries with greater numbers of older adults now being offered renal replacement therapy [1, 2]. Haemodialysis initiation can lead to significant functional [3] and cognitive decline in older adults [4]. Thus, more careful attention to haemodialysis prescription and treatment frequency is needed in this elderly patient group, aiming to preserve residual kidney function and prevent some of the detrimental effects associated with the standard paradigm of thrice weekly four-hour haemodialysis sessions. Similarly, the standard sessional Kt/Vurea clearance targets set by clinical guideline committees may not equally be applicable to all older adults now being treated with dialysis. In addition, some elderly patients offered dialysis may have limited life expectancy due to cancer or other terminal conditions, and decisions about dialysis schedules should be made jointly with an informed patient or their representatives, balancing the potential benefits of dialysis treatments with possible discomfort associated with transportation to and from dialysis centres and dialysis treatments.

In this review we discuss the limitations in generalisability of routine dialysis targets to older adults and discuss alternative parameters that can potentially be employed in routine clinical practice.

### Frequency of dialysis: should incremental dialysis be the first choice for elderly patients starting dialysis?

The 2015 update of the Kidney Disease Outcomes Quality Initiative (KDOQI) Clinical Practice Guidelines recommended that twice-weekly treatment be considered for patients with a residual native kidney urea clearance of more than 3 ml/min/1.73 m^2^ [5]. However, in clinical practice, as many centres do not routinely monitor residual kidney function, most patients on haemodialysis, especially those in countries with no resource limitations are generally started on thrice weekly dialysis [6].

The dose of dialysis that any patient requires should be tailored to their individual needs, particularly when considering older adults. There are two main elements to the dialysis prescription; firstly, clearance of the waste products of metabolism, and secondly preventing excessive hypervolaemia. Determining the dialysis dose in the older adult is complex and is affected by multiple factors including biological age, physical activity, frailty and functional status, severity of co-morbidities, and nutritional status.

Metabolic rate, energy expenditure, and muscle mass naturally decline with increasing age in healthy individuals, along with appetite and thirst sensation [7]. Active energy expenditure is reported to be lower with increasing age, clinical frailty score and co-morbidities [8]. Coupled with a reduced thirst drive and interdialytic weight gains, the generation rate of uremic toxins tends to be lower in older adults, and so many older patients do not require the same amount of dialysis compared to younger, physically active patients.

Thus, elderly and more frail patients in particular, require greater individualisation of their dialysis prescription, so that targets set are appropriate to their clinical condition, functional status, physical activity level, co-morbidities, stage of life and personal priorities (Table 1). Incremental dialysis (defined in this review as any haemodialysis prescription less than the standard paradigm of thrice weekly 4-h sessions) has many potential benefits and should be considered as the primary choice for most elderly patients initiating dialysis with residual kidney function. Observational studies have suggested that incremental dialysis may better preserve kidney function than the traditional thrice weekly approach [9], as repeated episodes of intradialytic hypotension may potentially lead to a more rapid loss of residual kidney function [10, 11]. A small prospective trial of incremental dialysis did not show any difference in the decline in residual kidney function compared to the standard thrice weekly paradigm [12]. Therefore, large prospective trials focusing on elderly patients are needed to definitively determine whether incremental dialysis leads to better preservation of residual kidney function, as residual kidney function has been reported to provide multiple benefits including better self-reported health-related quality of life, quicker post-dialysis recovery times [13], reduced inflammatory mediators and more effective anaemia management with reduction in iron and erythropoietin stimulating agent requirements [14].

**Table 1:** Comparing the fixed target approach with individualised management for dialysis in two older adults

		Patient A	Patient B
Characteristics			
Demographics		78 year old White male, weight 80 kg, body mass index 25.8 kg/m^2^	78 year old White male, weight 65 kg, body mass index 21.0 kg/m^2^
Cause of end-stage kidney disease		Vasculitis	Diabetes mellitus (DM)
Co-morbidities		Hypertension	Ischaemic heart disease, congestive heart failure, DM, hypertension, peripheral vascular disease
Residual kidney function		Unable to carry out 24-h urine collection because of work commitments	Incontinent, unable to carry out 24-h urine collection
Functional status		Walks 30 min every day, still works part time. Clinical frailty score^a^ 3. Loves to eat meat but has been restricting his intake because of kidney disease	Mostly sedentary. Clinical frailty score 5. Has experienced reduction in appetite with advancing kidney disease
Laboratory data (pre dialysis initiation)		Serum urea 38 mmol/L, creatinine 800 μmol/L, β2 microglobulin 15 mg/L,NT-proBNP 15000 pg/ml, albumin 38 g/L	Serum urea 25 mmol/L, creatinine 600 μmol/L. β2 microglobulin 15 mg/L, NT-proBNP 25000 pg/ml, albumin 30 g/L
Management			
Parameter	Standard recommendation	Suggested approach	Suggested approach
Dialysis prescription	Start dialysis 4 h 3 × week	Incremental dialysis twice weekly. Increase frequency of dialysis for clearance^b^ or volume triggers^c^	Incremental dialysis twice weekly. Increase frequency of dialysis for clearance^b^ or volume triggers^c^
		More likely to need longer dialysis sessions in view of active lifestyle and dietary protein intake	More likely to need more frequent dialysis due to volume triggers
Dietary recommendations	Protein intake 1–1.2 g/kg/day, dietary phosphate restriction, use of phosphate based binders	Follow guideline recommendations as patient has high dietary phosphate intake. Use phosphate binders as needed to achieve target phosphate levels	Non-restrictive diet. Allow patient higher salt intake to improve taste of food, consider nutritional supplementation
Access	Fistula first	Shared discussion about risks vs benefits of arterio-venous fistula (AVF) creation. Likely suitable for pre-emptive AVF creation 6–9 months prior to dialysis initiation	Shared discussion about risks vs benefits of AVF creation. Likely preferred access long term tunnelled catheter

*NT-proBNP* N-terminal pro–B-type natriuretic peptide

^a^Clinical frailty scale. https://www.bgs.org.uk/sites/default/files/content/attachment/2018-07-05/rockwood_cfs.pdf

^b^Clearance triggers for increasing frequency of dialysis: pre dialysis serum urea > 30 mmol/L, serum bicarbonate < 18 mmol/L despite oral bicarbonate supplementation, serum K > 6.5 mmol/L, Β2 microglobulin ≥ 30 mg/L

^c^Volume control triggers for increasing frequency of dialysis: urine output < 400 mL/day, interdialytic weight gain ≥ 4%, or NT-proBNP > 30,000 pg/ml

An important potential benefit of implementing an incremental dialysis approach is the effect on cognition. Patients treated with haemodialysis have a high prevalence of cognitive dysfunction [4, 15, 16]. The haemodynamic and osmotic changes during a haemodialysis session lead to both a decrease in cerebral perfusion and an increase in brain water content, with a resultant increased risk of brain damage, characterised by microvascular ischaemic changes on brain imaging [17–19]. Elderly patients are less likely to be able to compensate for the rapid decline in intradialytic systolic blood pressure due to impaired autonomic cardiovascular response, which, coupled with atheromatous and arteriosclerotic vascular disease, are more vulnerable to the sudden reductions in cerebral blood flow, with the combination of reduced cerebral perfusion and cerebral osmotic stress resulting in ischaemic brain white matter changes. It is well recognised that patients on haemodialysis have an increased risk of leukoaraiosis [20] and ischaemic stroke [21]. A rapid cognitive decline is particularly of concern in an elderly dialysis patient as it may lead to increased dependency and an increased risk of falls, as well as an inability for self-management, including compliance with medications. Reducing the frequency of dialysis can potentially reduce the repeated insults to the brain and decrease the rate of cognitive decline. Greater preservation of residual kidney function, as reported in observational studies of incremental dialysis, can potentially preserve the self-reported health quality of life and cognitive assessment scores of older patients treated with haemodialysis [22]. Thus, starting with incremental dialysis may have significant benefits with regard to preventing the rapid cognitive decline reported in studies of elderly patients treated with conventional thrice-weekly dialysis schedules.

In addition to more elderly, frail patients currently being offered haemodialysis, patients now generally also initiate dialysis earlier [23], and as such, the majority of elderly patients starting dialysis have residual kidney function. The KDOQI clinical guidelines do recommend that less than the standard thrice weekly 4-h dialysis sessions can be offered to patients with residual renal urea clearance > 3 mL/min/1.73 m^2^. However, elderly frail patients are less likely to be able to accurately carry out 24-h urine collections due to immobility, incontinence, and dementia [24, 25], and even if urine can be collected, residual renal urea clearance simply reflects glomerular clearance. While combining urea and creatinine clearance more closely resembles inulin clearance, the benefits of residual kidney function may not solely be due to glomerular filtration but might also reside with renal tubular secretion of protein-bound uraemic toxins and clearance of middle molecules. Although there are published algorithms combining residual renal urea clearance and dialyser sessional Kt/Vurea to provide an overall estimate of clearance, it must be remembered that 1 mL/min of dialyser urea clearance does not equate to 1 mL/min of residual renal urea clearance, and in particular 1 mL/min of combined urea and creatinine clearance [26, 27]. In addition, urine volume and urea concentration drop in the 24 h following a dialysis session, and then start to increase, so timing of urine collections can be a confounder when estimating residual renal urea clearance. Given the limitations of urinary collections and estimation of residual renal urea clearance, alternate parameters have been explored. Although there is currently no simple biomarker that can accurately be used as a surrogate of combined residual kidney function and dialyser clearance, β2 microglobulin (β2M), and trends in β2M are often used in clinical practice to guide dialysis prescriptions [28]. Beta trace protein and cystatin C have also been investigated as surrogate biomarkers for estimating residual kidney function [29, 30]. Using a combination of these markers may potentially allow more dialysis centres to offer elderly frail patients an incremental dialysis approach in the absence of reliable 24-h urinary collections.

Careful clinical assessment of volume status and adjusting post-dialysis target weights may help preserve residual kidney function to the same extent as using additional tools such as bioelectrical impedance analysis [31]. If the managing physicians prefer to use additional tools in volume management, then parameters like serum N-terminal pro–B-type natriuretic peptide (NT-proBNP), bioelectrical impedance analysis and lung ultrasound assessments can be utilised. Advances in dialysis machine technology enable the response to ultrafiltration to be reviewed with relative blood volume monitoring or changes in venous oxygen saturation for those dialysing with venous catheters. For the elderly dialysis patient, although trends in NT-proBNP, bioimpedance and lung ultrasound may aid clinical judgement, absolute values may not be so helpful when compared to those of younger patients due to underlying cardiovascular and respiratory diseases and changes in body composition with ageing. As such there remains a key role for regular clinical assessments, physical examination and review of dialysis charts when determining post-dialysis target weights.

Although most elderly patients may have lower interdialytic weight gains due to lower thirst drive and smaller appetite, attention should be paid to special groups of patients that may have lower cardiovascular reserve due to restrictive cardiomyopathies or reduced diastolic function, and are therefore more vulnerable to acute decompensation due to volume overload than patients with normal cardiac function. These patients may still need thrice weekly dialysis despite relatively lower interdialytic weight gains. Similarly, patients with severe valvular heart disease or pulmonary hypertension may be intolerant of rapid fluid removal and require longer or more frequent dialysis sessions to achieve clinically judged target weights.

**Fig. 1:**
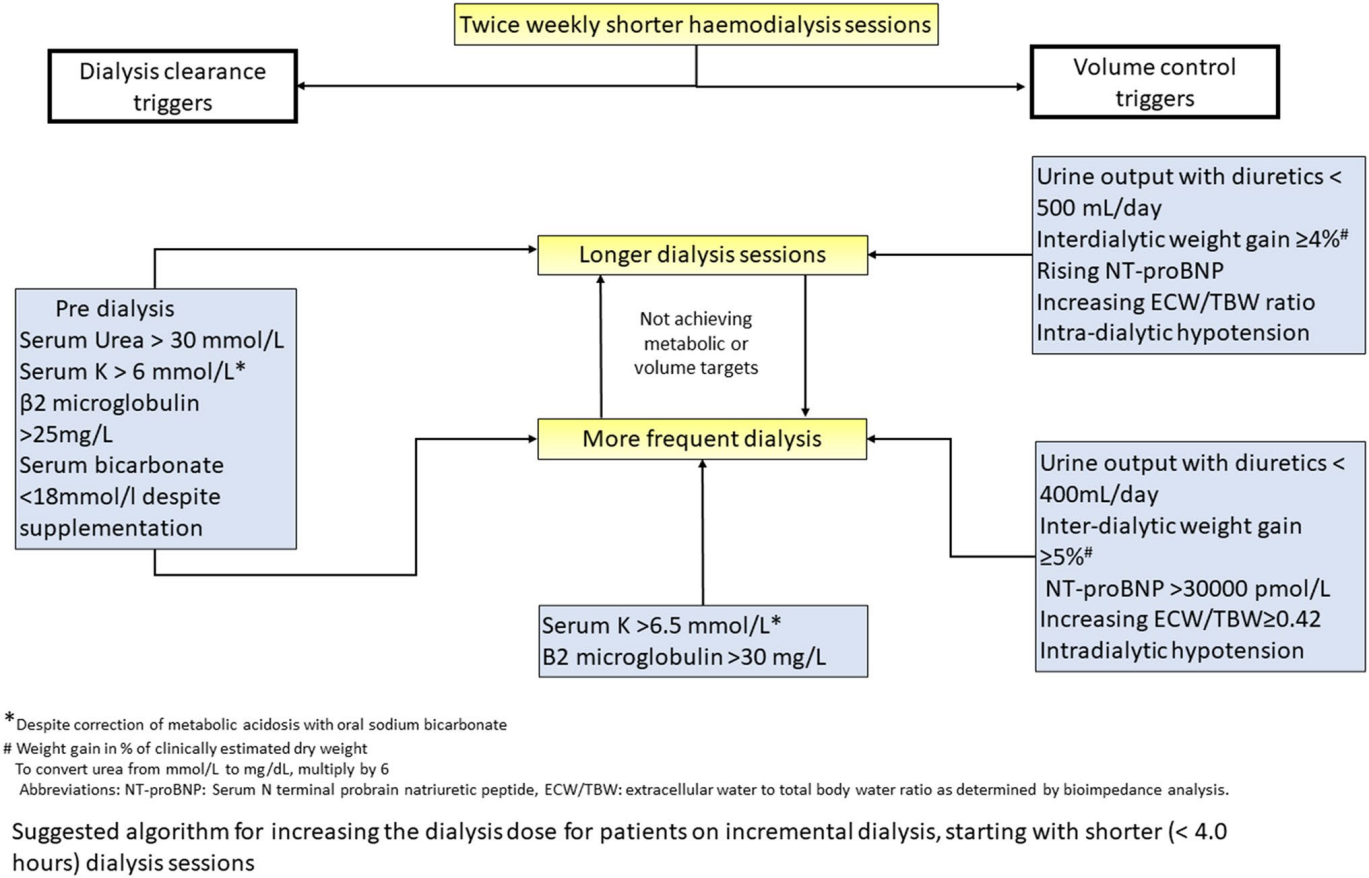
Clinical triggers when to consider either an increase in dialysis sessional duration or frequency

In summary, thrice weekly 4-h dialysis sessions should not be routinely prescribed as standard of care for older adults initiating dialysis, and individualised incremental dialysis regimens should be considered based on metabolic requirements, volume status and cardiovascular reserve. A suggested algorithm for increasing the dialysis dose for patients on incremental dialysis is presented in Fig. 1.

### KT/Vurea targets: do they equally apply in the elderly?

Traditionally, dialyser urea clearance has been used as a surrogate marker for the adequacy of dialysis treatments. To adjust for differences in patient size, urea clearance is indexed to the volume of urea distribution as Kt/Vurea, and as such Kt/Vurea is one of the most well-established parameters of dialysis adequacy and quality [32]. Numerous national and international clinical guidelines recommend a minimum Kt/Vurea target for each haemodialysis session [5, 33]. However, these guidelines do not consider the role of ageing or frailty in defining the minimum Kt/Vurea target. There are many reasons for the inaccuracies of Kt/Vurea calculations in elderly patients [34]. Most of them are related to the inaccurate assessment of the volume of urea distribution using the standard anthropometric measurements in the elderly, due to changes in body composition that naturally occur with ageing, accompanied by the physiological changes of ageing in terms of reduced appetite, thirst sensation and physical activity that affect the generation of uraemic toxins, including urea, and assumptions about renal handling of urea and water [35]. As such, for both elderly men and women the sessional Kt/Vurea appears to be much higher compared to a younger patient matched for gender, height, and weight [36]. This apparently higher dialyser Kt/Vurea clearance is due to the changes in body composition associated with advancing age, following loss of muscle mass and gain in fat weight and thus may not reflect an accurately delivered dialysis dose. In addition, the generation of waste products of metabolism is reduced due to the loss of metabolically active muscle [37] accompanied by decreased physical activity and appetite, thereby reducing the generation of toxins, and dialysis clearance requirements. Thus, the question arises as to the target sessional Kt/Vurea for the very elderly with reduced nutritional intake (Table 1), who may be living sedentary lives, but there is currently a lack of objective clinical trial data about dialysis adequacy threshold targets for this group.

The main proposition to support the continued use of dialyser Kt/Vurea as the measure of the adequacy of dialysis has been related to observational reports demonstrating an association between delivering lower sessional Kt/Vurea clearances and increased patient mortality, although in these reports lower Kt/Vurea was predominantly consequent on shorter dialysis sessions [38]. However, these findings have not been reproduced in the older age group. Indeed, a study from Spain reported that in a dialysis cohort with mean age of 70 years, those with higher sessional Kt/Vurea clearances had worse survival [39]. Moreover, recent studies, which have centred on patients rather than treatment deliverables, have shown that longevity is not the top priority for older adults, and that other outcomes, including the ability to maintain function, reducing pain and other symptoms are ranked as more important [40].

Despite its limitations, sessional Kt/Vurea clearance is relatively easy to measure, and a widely used tool in everyday clinical practice which can help guide management of dialysis treatment in older adults. Thus, when treating the very elderly, the limitations of KT/Vurea should be kept in mind when used as a measure of adequacy of dialysis, such that a lower sessional KT/Vurea in an older frail adult should not necessarily lead to an automatic increase in dialysis dose without considering residual kidney function and the individual preferences of the patient.

### Nutritional parameters in the elderly: easy to set but difficult to follow?

Protein energy malnutrition and sarcopenia are common problems encountered in elderly frail patients on haemodialysis, with a higher prevalence reported compared to the general population [41, 42]. Muscle mass generally declines with older age, but the term sarcopenia is usually used to indicate greater than age-expected loss of muscle mass. A concurrent protein energy wasting state may also exist if patients have lost fat weight and have lower serum albumin levels [43]. These disorders lead to greater morbidity including increased risk of falls, frailty, dependency, impaired quality of life as well as a higher mortality risk [44].

There are various national and international guidelines recommending protein and energy intake targets for dialysis patients [44–46], and these generally advise increased protein intake to compensate for protein loss during dialysis. The KDOQI guidelines recommend 1–1.2 g/kg protein intake per day to maintain a stable nutritional status in adults who are metabolically stable. However, striking a balance between following a sodium-, potassium- and phosphate-restricted diet and achieving these nutritional targets is difficult. Often the diet becomes repetitive and bland. Achieving these targets can be difficult for the older frail patient because of reduced appetite, limited access to freshly prepared food, long travel times and waits to and from the dialysis centre in view of transport requirements, and post-dialysis fatigue [46]. Therefore, less frequent dialysis, with less delivered dialysis may paradoxically improve the nutritional status of the frail elderly patient by allowing them to spend more time at home and limiting post-dialysis fatigue. In addition, although there may be some protein loss in urine, patients with residual kidney function generally have a higher dietary protein intake, which then drops as the residual function is lost [47]. Residual kidney function allows the patient a more liberal diet with greater food choices, so preservation of residual kidney function is important in maintaining nutritional intake in the elderly patient.

Appetite may additionally be reduced in elderly patients due to reflux oesophagitis and other gastrointestinal disorders, along with poor dental hygiene due to loosening of teeth associated with jawbone loss. In addition, dietary advice to follow a low sodium and phosphate diet, coupled with reduced taste and sense of smell often leads to a bland unappetising diet, thereby worsening the overall nutritional status [48]. The Kidney Disease: Improving Global Outcomes (KDIGO) guidelines recommend lowering elevated phosphate towards the normal range in patients on dialysis [49]. There are three main ways to reduce serum phosphate: dietary modification, phosphate binders and increasing the duration and frequency of the dialysis sessions. Restricting dietary phosphate generally also leads to a significant reduction in dietary protein intake, which, coupled with a naturally declining dietary intake with advancing kidney failure as well as age, can potentially pave the way for significant protein energy wasting. Moreover, the need for multiple phosphate binders can potentially add to the problem of polypharmacy and medication compliance in older adults. As such, an individualised approach to nutrition is therefore required for this group of patients rather than blanket target indices. Although more frequent nocturnal dialysis, and in particular the more frequent dialysis network nocturnal study group, demonstrated very good serum phosphate control in a younger patient cohort [50], it is less likely that extended dialysis sessions, especially if more frequent, would be suitable for the older more frail patient as it would most likely result in hypophosphataemia.

To prevent malnutrition, nutritional screening should be routinely incorporated into the care of elderly dialysis patients, so that patients at risk of further deterioration can be identified earlier and provided with appropriate support. There are various ways in which nutritional assessments can be performed. Several nutritional assessment scores, including the subjective global assessment, malnutrition inflammation score and geriatric nutritional risk index have been described in literature as screening tools for dialysis patients who are at nutritional risk [51] and potentially greater mortality [52, 53]. However, these scores are often time consuming and require dedicated professionals to conduct the assessments. In the absence of formal nutritional assessments, simple measures such as serial measurements of body weight, body mass index, physical examination by the supervising clinician and serum albumin values can provide useful insights about nutritional status over time. If feasible, serial assessments of muscle mass using non-invasive techniques including bioelectrical impedance assessment, anthropometry, and functional assessments of muscle strength using hand grip strength dynamometry may be used as adjuncts to clinical parameters in a nutritional screening programme for elderly frail dialysis patients [54].

Once patients at risk of deteriorating nutritional status are identified, they should be encouraged to follow a more liberal diet. This would mean taking away the typical low salt and low phosphate restrictions. The dialysis prescription should be reviewed to determine if there has been inadequate clearance of solutes, and whether increasing the amount of dialysis would potentially improve nutritional status or if there is another cause for poor appetite which can be effectively treated. If the decision is made to increase dialysis dose by increasing the frequency of dialysis, physicians should keep in mind that more frequent dialysis sessions may affect the patient’s appetite due to post dialysis fatigue and their inability to shop for and cook food. A time-based trial of increased dialysis dose and its effect on the overall nutritional parameters can help with this dilemma. Some of the other measures to potentially optimise the nutritional status in the elderly include dietary counselling [55], oral nutritional supplementation [56], and peri-dialytic oral—and in severe cases—intradialytic parenteral nutritional supplementation [57].

### Vascular access in the elderly: fistula first?

A good functioning vascular access is often considered the lifeline for the patient on haemodialysis. Generally, a native arteriovenous fistula is considered the preferred access for elective dialysis initiation because of its longer survival and lower complication rate. Many national and international guidelines recommend arteriovenous fistulas as the access of choice for patients initiating haemodialysis [58–60].

The “fistula first” approach may neither be optimal nor practical for an older patient on haemodialysis. The optimal timing of pre-emptive arteriovenous fistula creation in this group is not known. Many older patients lose kidney function more slowly than their younger counterparts and have a competing risk of mortality from other co-morbidities and therefore are more likely to die before benefiting from an arteriovenous fistula [61]. Elderly patients are more likely to have vascular calcification, and coupled with atheromatous vascular disease, many are not suitable candidates for a forearm fistula, and so they more often have a brachial fistula created, which is more likely to have higher flows causing a greater cardiac shunt. Thus, a brachial fistula may induce adverse cardiovascular consequences in elderly patients with calcific aortic stenosis, and in those with cardiac failure, particularly with diastolic dysfunction. Fistula maturation, and primary and secondary patency rates show a decreasing trend with advancing age [62–64]. While central venous catheters are the least preferred vascular access option for haemodialysis, they tend to be associated with a lower risk of catheter-related bloodstream infections in the elderly [65].

Therefore, it would be appropriate to adopt an individualised approach to the choice of vascular access in older patients on haemodialysis, especially for the frail octa- and nonagenarian with multiple co-morbidities. Factors that should be considered in shared discussion with the patient include patient preference, choices about quality of life, such as pain from cannulation, life expectancy, co-morbidities, and individual centre experience (Table 1). If the decision is made to create an arteriovenous fistula, a more liberal use of proximal access sites can be considered provided the patient has adequate cardiovascular reserve [66].

## Conclusion

The demographics of the dialysis population in Western Europe and North America have changed over the last decade, with increasing numbers of elderly frail patients now being offered dialysis. This change in demographics, coupled with more patients now initiating dialysis with greater amounts of residual kidney function demands reappraisal of clinical guidelines developed from the 1990s since the currently advised dialysis prescription, dialyser clearance, nutritional and vascular access targets may not always be appropriate to guide the clinical management of older frail patients treated with haemodialysis. Instead of the standard thrice weekly 4-h dialysis sessions, an individualised prescription taking into consideration incremental dialysis may potentially help to preserve residual kidney function and provide additional benefits in terms of quality of life for the older patient. Thus, a more holistic approach should be considered, taking into account residual kidney function, markers of dialyser clearance, volume control, haemodynamic stability, post-dialysis fatigue and patient preferences. Although this current review focuses on haemodialysis in older adults, these principles can be applied to individuals initiating any form of dialysis. Dietary intake is often reduced in the older patient thus nutritional parameters should be closely monitored and if needed, liberalisation of the standard low salt, potassium and phosphate diet should be allowed to encourage adequate caloric and protein intake. Although a fistula may be the preferred vascular access option, arteriovenous fistula creation may not always be practical due to vascular or cardiac disease, and so dialysis catheters then become the access of choice, especially in the frail elderly patient with a limited life span. Blindly following clinical guideline-recommended targets in the elderly may potentially lead to adverse consequences, which are summarised in Table 2. Therefore, a more patient-oriented approach would be more appropriate in deciding on vascular access options, and dialysis prescriptions rather than prescriptive targets.

**Table 2:** Summary of effects of standard dialysis practices on elderly patients

Parameter	Physiological differences in the elderly	Potentially deleterious effects
Dialysis frequency of three times a week	Lower muscle mass, energy expenditure and interdialytic weight gains potentially allowing an incremental schedule More predisposition to hypotension given impaired autonomic response Prolonged recovery time Limited mobility, reliance on others for travel to and from dialysis centre	Increased risk of intradialytic hypotension with potentially accelerated loss of residual kidney function (RKF) and its benefit More rapid decline in cognitive function Decreased quality of life (QOL) Post-dialysis fatigue impacting on nutritional intake with increased risk of sarcopenia and protein energy wasting Increased caregiver burden
Target of a minimal Kt/Vurea as a marker of dialysis dosing	Erroneous values of Kt/V when using Watson equation to estimate total body water volume as a result of loss of muscle mass with ageing	Increase in dialysis frequency/duration to achieve fixed targets leading to more rapid loss of RKF
	Limited evidence about utility of Kt/Vurea in elderly	More time spent in dialysis units than with family and friends
Recommendations on low salt, low potassium and low phosphate diet	Reduced taste and sense of smell	Protein energy malnutrition
	Poor appetite due to reflux oesophagitis and other gastrointestinal disorders	Sarcopenia
	Low muscle mass at baseline predisposing to malnutrition	Increased risk of falls and fractures
Arteriovenous fistula (AVF) as access of choice	High burden of atherosclerotic disease	Higher likelihood of primary failure
	Increased risk of underlying myocardial or cardiac valvular heart disease	Increased risk cardiac decompensation with higher flow brachial fistula
	Higher likelihood of vascular steal syndrome	Pain related to needling affecting QOL
	Higher cardiac risk for surgery	Stress of AVF surgery
	Competing risk factors for mortality	Higher chances of death from other causes before the AVF is used
